# Cardiotoxicity of Zebrafish Induced by 6-Benzylaminopurine Exposure and Its Mechanism

**DOI:** 10.3390/ijms23158438

**Published:** 2022-07-29

**Authors:** Mengying Yang, Jialu Luan, Yixin Xu, Chengtian Zhao, Mingzhu Sun, Xizeng Feng

**Affiliations:** 1 State Key Laboratory of Medicinal Chemical Biology, The Key Laboratory of Bioactive Materials, College of Life Science, Ministry of Education, Nankai University, Tianjin 300071, China; 18435144936@163.com (M.Y.); 2120211215@mail.nankai.edu.cn (J.L.); 2120201098@mail.nankai.edu.cn (Y.X.); 201730832033@mail.scut.edu.cn (C.Z.); 2 The Institute of Robotics and Automatic Information Systems, Nankai University, Tianjin 300071, China

**Keywords:** zebrafish, 6-benzylaminopurine, cardiotoxicity, HPA axis activity, inflammation

## Abstract

6-BA is a common plant growth regulator, but its safety has not been conclusive. The heart is one of the most important organs of living organisms, and the cardiogenesis process of zebrafish is similar to that of humans. Therefore, based on wild-type and transgenic zebrafish, we explored the development of zebrafish heart under 6-BA exposure and its mechanism. We found that 6-BA affected larval cardiogenesis, inducing defective expression of key genes for cardiac development (*myl7*, *vmhc*, and *myh6*) and AVC differentiation (*bmp4*, *tbx2b*, and *notch1b*), ultimately leading to weakened cardiac function (heart rate, diastolic speed, systolic speed). Acridine orange staining showed that the degree of apoptosis in zebrafish hearts was significantly increased under 6-BA, and the expression of cell-cycle-related genes was also changed. In addition, HPA axis assays revealed abnormally expressed mRNA levels of genes and significantly increased cortisol contents, which was also consistent with the observed anxiety behavior in zebrafish at 3 dpf. Transcriptional abnormalities of pro- and anti-inflammatory factors in immune signaling pathways were also detected in qPCR experiments. Collectively, we found that 6-BA induced cardiotoxicity in zebrafish, which may be related to altered HPA axis activity and the onset of inflammatory responses under 6-BA treatment.

## 1. Introduction

The environment is closely related to human production and life, and environmental pollution has become one of the hot topics in recent years. Therefore, pesticides and their residues that are highly related to human diet are also increasingly aroused by people’s warnings. 6-Benzylaminopurine (6-BA), a major plant growth regulator, plays a major role in plant growth promotion and fruit and vegetable preservation [1]. There are data showing that 6-BA is less toxic, suggesting that it has less effect on the organism [1]. However, this may ignore the fact that pesticides are used in production at much higher than recommended concentrations and often in excess. In addition, studies have found that 6-BA may have specific organ-targeted toxicity, and it is not easily degraded in moist soil and water [2]. At present, many countries have clearly stipulated the use of 6-BA and its residue limits. Based on previous studies, we found that 6-BA can induce dose-dependent toxicity, cause stage developmental malformations in zebrafish, and lead to abnormal larval behavior [3], which further illustrates the harm of 6-BA, at least to aquatic organisms.

Many studies on acute toxicology are based on zebrafish models. The zebrafish (*Danio rerio*), as a vertebrate, has a high degree of genome homology with humans, which makes it an invaluable model for developmental, neurological, and toxicological studies [4]. The heart is the first organ in vertebrate phylogeny and plays a vital role in the organism. The whole body of a zebrafish is transparent in the early development stage, which is convenient to observe the development of various organs under drug exposure. Therefore, to further explore the effects on the heart under 6-BA exposure, we chose zebrafish as the experimental subject.

The hypothalamic–pituitary–adrenal (HPA) axis is a body system that involves the hypothalamus, pituitary, and adrenal glands and is activated in response to environmental stress [5]. Specifically, the hypothalamus secretes corticotropin-releasing hormone (CRH), which in turn acts on the pituitary to release adrenocorticotropic hormone (ACTH). ACTH then acts on the adrenal gland to secrete glucocorticoid (GC), which modulates HPA axis activity through the glucocorticoid receptor (GR or nr3c1) [6]. The HPA axis is an important aspect of the neuroendocrine system because it controls stress responses and regulates many physical activities. The ability of GCs to alter neuroendocrine balance, metabolism, sympathetic activity, and immune function implies that systemic maladaptation to stress may underlie the increased susceptibility of the cardiovascular system to pathological pathogenesis [7]. Alterations in the glucocorticoid receptor (GR)-mediated stress response pathway have been found to contribute to developing cardiac dysfunction, and the antagonistic interaction between GR and the cytokine interleukin 4 (IL-4) is a key factor in cardiac development [8]. Furthermore, chronic blood pressure elevation and other endocrine dysfunctions are one of the major causes of adverse cardiometabolic events caused by altered HPA axis function [9,10]. Therefore, to better explore whether 6-BA affects cardiac development and its underlying mechanisms, we also explored the HPA axis and immune signaling pathways, which are critical for coordinating cardiac physiological growth.

In this experiment, zebrafish embryos were used as the research object to study the toxicological effects of 6-BA exposure through acute exposure treatment. The cardiac morphology, cardiac development and function, and cell apoptosis in the early embryonic development of zebrafish were counted, and the levels of inflammation-related factors and HPA activity-related genes and hormones were also detected. In addition, thigmotaxis behavior, as a behavioral indicator of early zebrafish development and a cascade of altered HPA activity, was further explored. These results were used to assess the effects of acute 6-BA exposure on early cardiac development in aquatic vertebrates.

## 2. Results

### 2.1. 6-BA Induced Cardiac Developmental Malformations in Zebrafish

**Figure 1:**
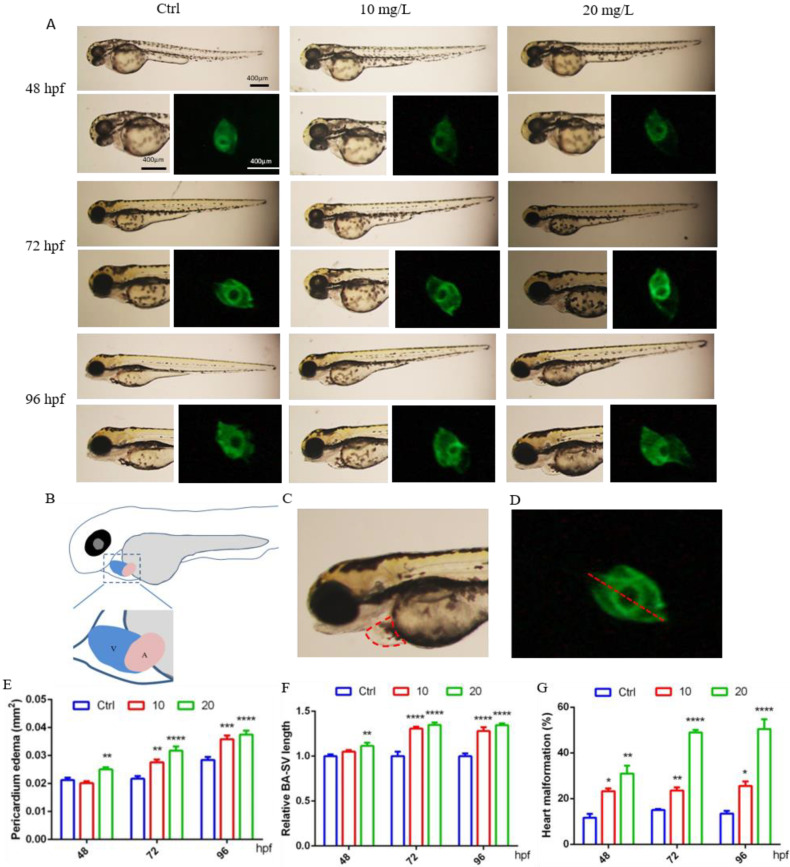
6-BA induced malformation of zebrafish heart development. (**A**) Cardiac developmental status and fluorescence images of zebrafish larvae at different stages under 6-BA exposure. (**B**) Schema of the atria and ventricular position. (**C**) Schematic diagram of measurement area of pericardial edema area. (**D**) Representative diagram of the measured BA–SV length. (**E**–**G**) Statistical analysis of pericardial edema area (**E**), BA–SV distance (**F**), and cardiac malformation rate (**G**) in zebrafish over time. Values are expressed as mean ± standard error (SEM). N = 3. * *p* < 0.05, ** *p* < 0.01, *** *p* < 0.001, **** *p* < 0.0001.

It was observed that at 48 hpf, the hearts of control larvae circled into an S shape, with partially overlapping atria and ventricles. In contrast, some zebrafish exposed to 6-BA exhibited cardiac developmental malformations, such as increased pericardial edema and linearization of the relative position of the atrium and ventricle, which also resulted in a significant increase in the BA–SV distance in larvae (Figure 1A). Based on these two parameters, we also counted the rate of cardiac malformation in zebrafish. Through data analysis, we found that the pericardial edema area and BA–SV distance in the 20 mg/L 6-BA group were greater than those in the 10 mg/L 6-BA group, especially in the statistics of cardiac malformation rates (Figure 1B–D). Additionally, the toxicity of 6-BA continued to increase with the extension of development time, and it was more harmful to zebrafish at 72 and 96 hpf. Further, the pericardial edema area, BA–SV distance, and cardiac malformation rate were significantly different from those in the control (Figure 1A). This indicated that 6-BA had significant cardiotoxicity in zebrafish in a time- and concentration-dependent manner.

### 2.2. 6-BA Affected Zebrafish Cardiac Function and Gene Expression

**Figure 2:**
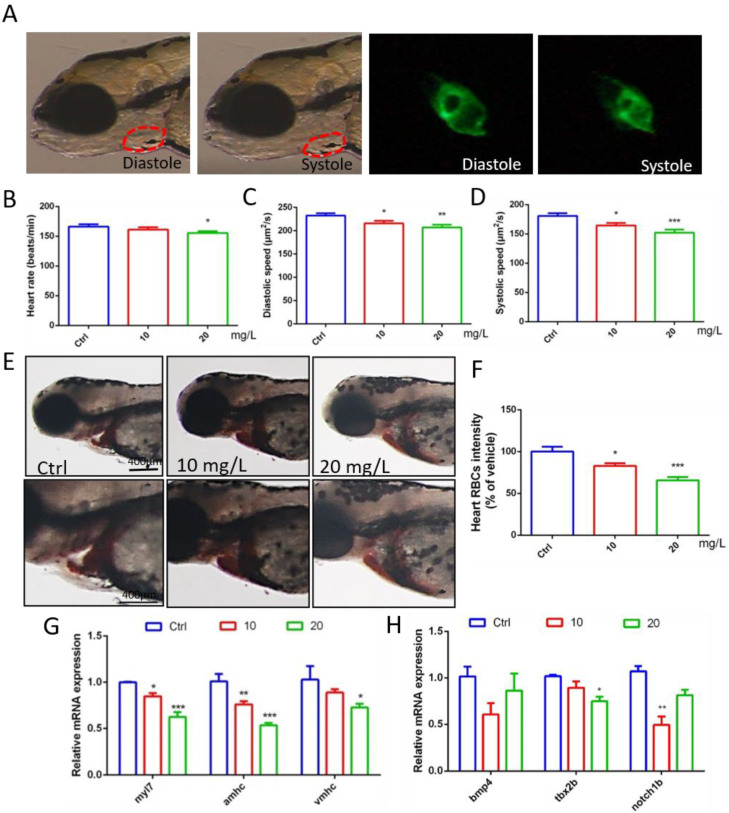
Abnormal gene expression and cardiac dysfunction in zebrafish exposed to 6-BA at 72 hpf. (**A**) Diastolic and systolic states of larval hearts under bright field and green fluorescence. (**B**–**D**) Statistical results of larval cardiac function parameters, including heart rate, diastolic velocity, and systolic velocity. (**E**) Image of zebrafish heart after o-dianisidine staining. (**F**) Quantitative analysis of cardiac staining results. (**G**) Expression of the myocardial regionalization marker genes *myl7*, *amhc*, and *vmhc* in zebrafish. (**H**) Expression levels of the atrioventricular canal (AVC) marker genes *bmp4*, *tbx2b*, and *notch1b* in zebrafish. Values are expressed as mean ± standard error (SEM). N = 3. * *p* < 0.05, ** *p* < 0.01, *** *p* < 0.001.

Through video processing and image analysis, we found that 6-BA had little effect on the heart rate of zebrafish when they developed to 72 hpf, the statistical results of the 10 mg/L 6-BA group were similar to the control, and 20 mg/L only induced a small decrease in the heart rate of larvae (Figure 2B, Supplementary Video S1). However, the diastolic and systolic velocities of zebrafish under 6-BA treatment were significantly reduced compared with controls, especially in the 20 mg/L 6-BA group, indicating that larval cardiac function was attenuated under 6-BA (Figure 2C,D, Supplementary Video S2). In addition, staining results showed that the number of erythrocytes in the heart of zebrafish was significantly reduced with the appearance of cardiac malformations (Figure 2E). The results of the quantitative analysis were consistent with the image observations. Compared with the control, the cardiac erythrocyte intensity of zebrafish in the 6-BA group gradually decreased with increasing exposure concentration (Figure 2F). Meanwhile, qPCR results showed that exposure to different concentrations of 6-BA induced a decrease in the mRNA expression of the embryonic hemoglobin genes *hbae3* and *hbbe1*, with significant differences compared with the control group (Figure S1). In addition, we found that blood flow rate decreased in larvae under 6-BA exposure (Figure S2). This showed that 6-BA reduced red blood cell influx into the heart in a dose-dependent manner, impairing blood circulation in zebrafish embryos.

To better understand cardiotoxicity in developing zebrafish exposed to 6-BA, we also performed experiments at the molecular level. qPCR detection revealed that the expression of the myocardial regionalization marker genes *myl7*, *vmhc*, and *amhc* decreased in a concentration-dependent manner (Figure 2G). The molecular-level differences caused by 6-BA suggest a collapse in cardiac development, which may be responsible for cardiac morphological and functional abnormalities. In addition, the expression levels of the atrioventricular canal (AVC) marker genes *bmp4*, *tbx2b*, and *notch1b* were also significantly reduced (Figure 2H), suggesting that AVC differentiation failure due to 6-BA toxicity affects cardiac development.

### 2.3. 6-BA Induced Larval Cardiac Apoptosis and Affected Cycle-Related Gene Expression

**Figure 3:**
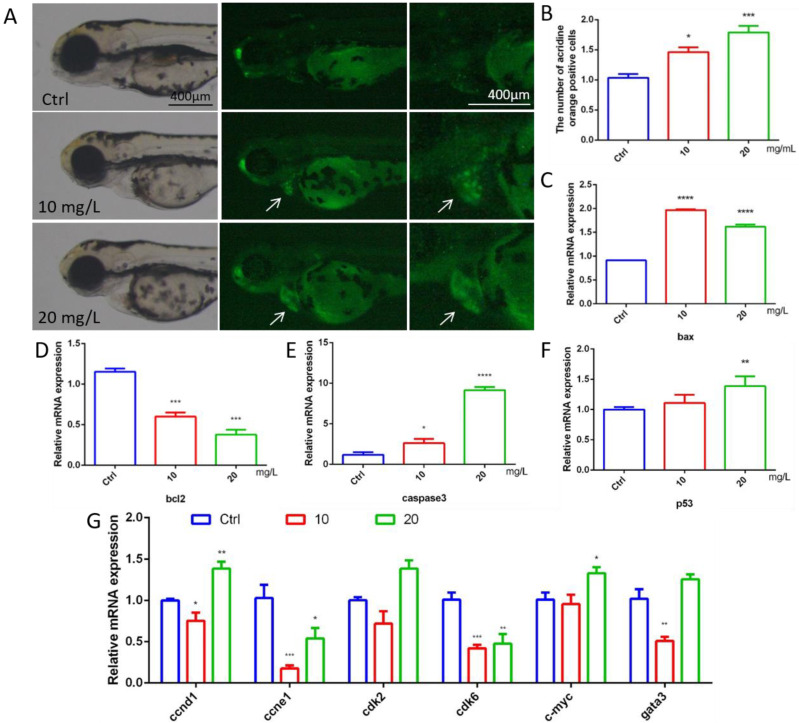
Apoptosis assessment and expression of cycle-related genes in 72 hpf zebrafish. (**A**) Fluorescence image of zebrafish after acridine orange staining under 6-BA treatment. (**B**) Quantitative statistics of apoptotic cells in larval hearts after acridine orange staining. Apoptotic cells appear as bright green spots. (**C**–**F**) The mRNA expression levels of the apoptosis-related genes *bax*, *bcl2*, *caspase3*, and *p53*. (**G**) Expression of cell-cycle-related genes, including *ccnd1*, *ccne1*, *cdk2*, *cdk6*, *c-myc*, and *gata3*. Values are expressed as mean ± standard error (SEM). N = 3. * *p* < 0.05, ** *p* < 0.01, *** *p* < 0.001, **** *p* < 0.0001.

To explore whether 6-BA affects cardiac apoptosis in zebrafish, we performed acridine orange staining experiments. Cardiac apoptotic cells appear as bright green spots. Compared with the control, the fluorescence brightness of larval hearts in the 10 mg/L6-BA group was enhanced, and the number of green spots increased significantly. However, the degree of heart apoptosis in the 20 mg/L treatment group was more obvious, and the picture showed bright green (Figure 3A). The quantification results of apoptotic cells were consistent with the picture observation, and the relative positive apoptotic cell number in the larval hearts gradually increased with the increasing concentration of 6-BA treatment (Figure 3B). The Bcl-2 protein family composed of proapoptotic members, such as *bax*, and antiapoptotic factors, such as *bcl2*, plays a key role in physiological processes. The study found that the mRNA level of *bcl2* in zebrafish decreased under 6-BA, while the expression levels of the proapoptotic genes *bax* and *caspase3* gradually increased (Figure 3C–E). In addition, the expression of the *p53* gene was induced by 6-BA (Figure 3F).

6-BA-induced apoptosis was supported by the mRNA levels of cell-cycle-related genes (Figure 3G). The expression of the cell cycle activation genes *ccne1* and *cdk6* was significantly reduced compared with the control, with statistically significant differences. The expression of *ccnd1* and *cdk2* was decreased in the 6-BA exposure group at 10 mg/L. Interestingly, the expression of the cell cycle activation genes *ccnd1* and *cdk2* and the mitogenic gene *c-myc* was increased in the 6-BA exposure group at 20 mg/L. The expression of *gata3*, a transcription factor that regulates a variety of physiological processes, also decreased and then increased with an increasing treatment dose.

### 2.4. The Effect of 6-BA on Inflammatory Factors

**Figure 4:**
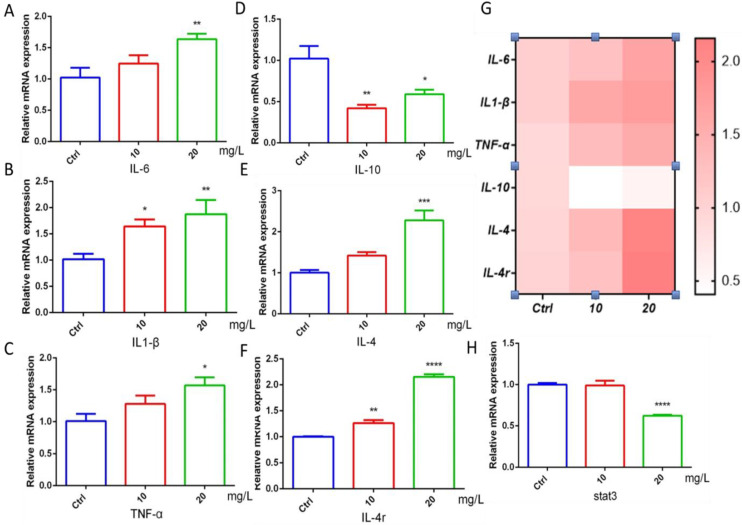
6-BA induced inflammation in zebrafish at 72 hpf. (**A**–**C**) Expression of IL-6, IL1-β, and TNF-α, the hallmark proinflammatory factors of inflammatory response in zebrafish treated with 6-BA. (**D**–**F**) The mRNA levels of the landmark anti-inflammatory factors IL-10, IL-4, and receptor IL-4r in zebrafish. (**G**) Heatmap of inflammatory-response-related genes. Red indicates higher values than the control, and darker colors indicate larger values compared with the control. (**H**) Relative mRNA expression of *stat3*, a downstream target gene of IL-4. Values are expressed as mean ± standard error (SEM). N = 3. * *p* < 0.05, ** *p* < 0.01, *** *p* < 0.001, **** *p* < 0.0001.

The occurrence of inflammatory response is closely related to cardiac function. The study found that the gene mRNA expression of the proinflammatory factors IL-6, IL1-β, and TNF-α in the 6-BA exposure group was higher than that in the control group, with a gradient increase (Figure 4A–C). The mRNA levels of IL-10, a marker of inflammatory responses, were induced to decrease, but increased in the 20 mg/L6-BA group (Figure 4D). Another anti-inflammatory factor, IL-4, and its cognate receptor IL-4r were induced by 6-BA, especially in the 20 mg/L 6-BA group (Figure 4E,F). These changes are also visualized in the heatmap (Figure 4G). The target gene Stat3 acts downstream of the IL-4 factor. IL-4 can alter zebrafish cardiac mitotic activity by regulating the Stat3 activation and transcription of genes that regulate cell cycle progression. qPCR showed that the Stat3 transcript level in the 10 mg/L6-BA exposure group was no different from that in the control, while 20 mg/L6-BA induced a significant decrease in its expression (Figure 4H). Overall, inflammation-related genes showed large differences due to 6-BA exposure, which may be associated with the induction of substantial toxicity, such as cardiotoxicity.

### 2.5. 6-BA Affected HPA Axis Gene Expression and Hormone Levels in Zebrafish

**Figure 5:**
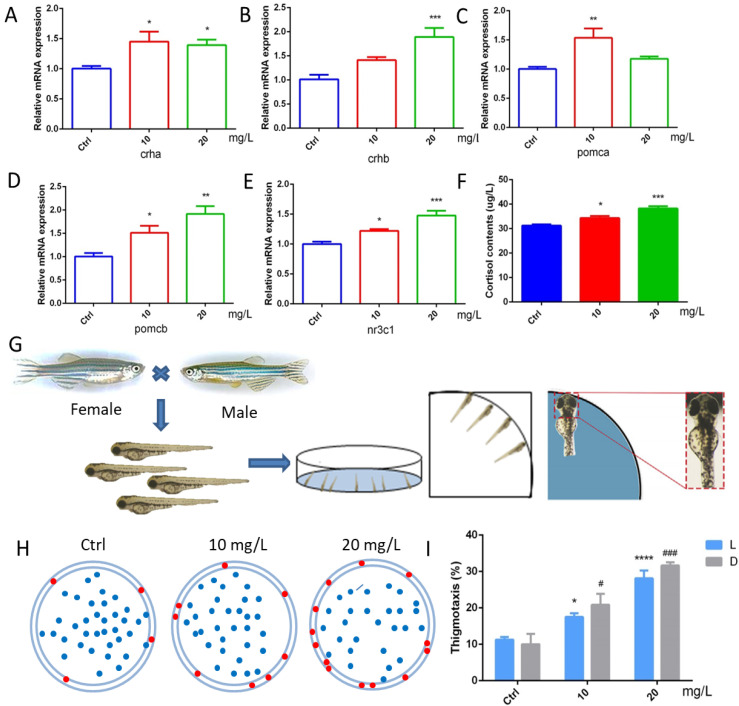
6-BA treatment affected HPA axis activity and thigmotaxis behavior in zebrafish. (**A**–**E**) Expression patterns of the HPA axis genes *crha* (**A**), *crhb* (**B**), *pomca* (**C**), *pomcb* (**D**), and *nr3c1* (**E**) in larval zebrafish after 6-BA exposure. (**F**) The effect of 6-BA on HPA axis hormone (cortisol) levels in larval zebrafish. (**G**) Zebrafish exhibited thigmotaxis under 6-BA stimulation, and larvae with only their heads in contact with the petri dish were defined as having positive thigmotaxis. (**H**) Overall distribution of zebrafish larvae in petri dishes under different treatments. Larvae exhibiting thigmotaxis are indicated by red dots on the edge of the petri dish, and the rest are indicated by blue dots. (**I**) Statistics of zebrafish larvae thigmotaxis under different treatments. Values are expressed as mean ± standard error (SEM). N = 3. * *p* < 0.05, ** *p* < 0.01, *** *p* < 0.001, **** *p* < 0.0001, # *p* < 0.05, ### *p* < 0.001.

To further explore the effect of 6-BA on HPA axis activity, we performed qRT-PCR analysis and determination of cortisol content (Figure 5A–F). We found that the expression of *crha* was induced by 6-BA at 3 dpf. The *crhb* gene level in the 6-BA group was increased compared with the control, especially in the 20 mg/L6-BA group; the difference was particularly significant. Transcript levels of *pomca* were higher in the 10 mg/L 6-BA group, but not different from the control in the 20 mg/L group. While the expression of its homolog *pomcb* gradually increased with the increase in exposure dose. The expression of *nr3c1*, a receptor for glucocorticoids that regulates the activity of the HPA axis, was also induced by 6-BA, with significant differences compared with controls. With these data, the HPA axis gene expression pattern of zebrafish larvae was greatly affected by 6-BA exposure. In addition, ELISA results showed that cortisol levels were significantly increased after exposure to 6-BA (Figure 5F), with a similar trend to its receptors. Collectively, the above data suggest that 6-BA alters HPA axis activity in larval zebrafish.

### 2.6. 6-BA Induced Anxiety-like Behavior

Zebrafish have well-defined behavioral indicators, such as exercise, anxiety, and depression. Thigmotaxis is a typical representative of anxious behavior in larval zebrafish. The analysis found that the zebrafish in the control group were evenly distributed in the dish, with only a few tending to the edge of the dish. However, a higher number of larvae in the 10 mg/L 6-BA-treated group than the control moved to the marginal area, and this situation was more pronounced in the 20 mg/L 6-BA exposure group, indicating that higher concentrations of 6-BA had a greater effect on its behavior (Figure 5H). In addition, we found that the thigmotaxis behavior of zebrafish after 6-BA exposure increased with increasing concentrations in both light and dark, and the thigmotaxis was more pronounced under dark conditions compared with controls (Figure 5I). Overall, 6-BA exposure altered zebrafish thigmotaxis and induced anxiety-like behaviors in larvae.

## 3. Discussion

The zebrafish is known to be a good vertebrate model for studying organogenesis and developmental toxicity. After fertilization, zebrafish develop rapidly into embryos, with most organ primordia established within the first 24 h. About 2 days after fertilization (dpf), the embryo hatches from the chorion and reaches the larval stage at about 3 dpf, when most of the internal organs have matured [11]. The heart is the first organ in an organism to form, mature, and function [12]. Although zebrafish have only two cardiac chambers, they exhibit many patterns during cardiogenesis that resemble common vertebrates and humans [13]. Pesticide pollution has attracted more and more attention because of its harmfulness. As one of the earliest synthetic plant hormones, 6-benzylaminopurine (6-BA) is widely used for its growth-promoting and fresh-keeping effects. However, 6-BA is often used in excess and at higher than recommended concentrations, which may be potentially harmful to the environment and organisms. So far, no conclusions can be drawn about the safety of using 6-BA in agricultural production. Our previous study found that 6-BA can affect the development and behavior of zebrafish embryos and larvae, and showed strong toxicity with increasing exposure time and concentration, which aroused our vigilance. Therefore, to further explore the toxicity of 6-BA and its mechanism, we evaluated the cardiac development of larval zebrafish treated with 6-BA.

Cardiogenesis is a complex process involving cell specification and differentiation as well as tissue morphogenesis and remodeling [14]. Spatial and temporal coordination between these processes is a prerequisite for the heart to maintain functional integrity throughout development. Observations of zebrafish heart morphology suggest that larval cardiac circularization is abnormal after 6-BA exposure. With the increase in exposure concentration and the prolongation of treatment time, the area of pericardial edema in zebrafish gradually increased, and the degree of linearization of larval heart was more obvious. Cardiac cyclization is an important period in cardiac morphogenesis. When zebrafish embryos develop to 24 hpf, the heart tube is asymmetrically elongated, the cardiac looping is S-shaped, and the atria and ventricles are gradually formed [15,16]. Thus, the observed cardiac malformations suggest that 6-BA may impair the process of cardiac formation in zebrafish, affecting chamber differentiation. *Amhc* and *vmhc* are the earliest marker genes to label the atrium and ventricle of the heart [17,18], respectively. *Myl7*, also known as *cmlc2*, is expressed in the atrium and ventricle, and is closely related to various physiological processes, such as cardiomyocyte proliferation, cardiac contraction, and myofibril assembly [19]. Significant downregulation of *myl7*, *amhc*, and *vmhc* expression was observed, which may be related to the various cardiac defects observed as well as pericardial edema and subsequent cell death [20]. Differentiation of the atrioventricular canal (AVC) is a key process in heart valve formation. The AVC separates the atria and ventricles and creates the endocardial cushion, which then contributes to the development of the heart valves and diaphragm. Restricted Bmp and Notch signaling is critical for AVC formation to promote glial formation, epithelial–mesenchymal transition, and AVC patterning [21]. We found that *bmp4*, *notch1b*, and another marker expressed in the myocardium, *tbx2b*, were downregulated upon 6-BA exposure, suggesting that 6-BA interferes with the molecular conditions required for atrioventricular valve formation in AVC. Developmental defects caused by inappropriate AVC formation have devastating consequences in affected individuals [22]. We speculate that the formation of cardiac developmental malformations is largely related to the abnormal expression of these development-related genes, which ultimately affects individual development. In addition, the statistical analysis of cardiac function parameters showed that the heart rate of zebrafish was not greatly affected by 6-BA, but the diastolic and systolic velocity of the heart were significantly reduced, indicating that the cardiac function of zebrafish was weakened under 6-BA, especially in the high concentration group. Together, zebrafish cardiac morphological and functional alterations suggest that acute 6-BA exposure induces cardiotoxicity by retarding cardiac development and attenuating cardiac function. Blood vessels develop after the formation of the heart and continue to form the cardiovascular circulatory system [23]. The reduction of erythrocytes in larval hearts after staining indicated impaired blood circulation in zebrafish, further demonstrating that 6-BA affects cardiac development. Taken together, these results suggest that the heart may be a target organ for acute 6-BA exposure-induced developmental toxicity.

Apoptosis is an important and irreversible damage in biological, physiological, and pathological processes [24], and apoptosis is widely used in the development and homeostasis of tissues and organs. Cardiomyocyte apoptosis is the main cause of progressive heart failure [25]. Acridine orange is a cell-permeable fluorescent dye that binds to the nucleic acids of dying cells, resulting in changes in spectral emission [26]. It was observed that the number of apoptotic points increased significantly in the 6-BA-treated group, the heart region showed bright green fluorescence, and the transcription of apoptosis-related genes (*bax*, *bcl2*, *caspase3*, *p53*) was induced to change. The balance between pro- and antiapoptotic protein regulators is a critical point in determining whether cells undergo apoptosis. Combined with the staining results, the above data indicated that 6-BA induced apoptosis in zebrafish. When DNA damage or other exogenous stress stimuli occur, the cell cycle repairs it to the normal stage of genome replication. However, dysregulation of multiple cell cycle regulators, including the cyclin/cdk complex, induces cell cycle arrest and cellular senescence, leading to apoptosis [27]. Results at the transcriptional level of cell-cycle-related genes support an apoptotic role for 6-BA in developing zebrafish. Transcription of the cell cycle activation genes *ccnd1*, *ccne1*, *cdk2*, *cdk6* was blocked. The increased expression of the cell cycle activation genes *ccnd1* and *cdk2* and the mitogenic gene *c-myc* in the 6-BA exposed group at 20 mg/L was likely due to the compensatory response of the organism after the effect of 6-BA on the cell cycle of zebrafish larvae. These data suggest that 6-BA treatment induces apoptosis during early stages of zebrafish development, which may be affected by cell cycle dysregulation, ultimately impairing cardiac development.

Altered phenotype and function of immune cells underlie adverse cardiovascular remodeling [28]. Studies have found that IL-4 signaling regulates cardiomyocyte mitosis during development [8]. Dihydrotanshinone I (DHT) improves cardiac function and reverses doxorubicin-induced cardiotoxicity by inhibiting M1 macrophage activation and excessive release of proinflammatory cytokines in vivo and in vitro [29]. In addition, two determinants and possible therapeutic targets of anthracycline cardiotoxicity have been reported, and inflammation is one of them [30]. In our experiment, the mRNA levels of the proinflammatory factors IL-6, IL1-β, and TNFα in zebrafish were upregulated by 6-BA, and the transcription of the anti-inflammatory factors IL-10 and IL-4 and the receptor IL-4r were also changed to varying degrees, indicating that there was a serious inflammatory response in zebrafish treated with 6-BA. The target gene *stat3* is activated by the combination of IL-4 and IL-4r [31], and is a common target of GR and IL-4. Further investigation revealed that the *stat3* gene level was downregulated in zebrafish under 6-BA exposure, and transcription of the stat3-targeted mitogenic genes *c-myc* and *gata3* was also induced by 6-BA. Changes in downstream genes of cytokines further prove that 6-BA induces inflammatory responses, which may have negative effects on cardiogenesis. Inflammatory responses can disrupt the activities of key factors in cardiac development, leading to the failure of early cardiac formation [32], so the inflammatory responses induced by acute exposure to 6-BA may contribute to its induced cardiac morphological and functional abnormalities.

**Figure 6:**
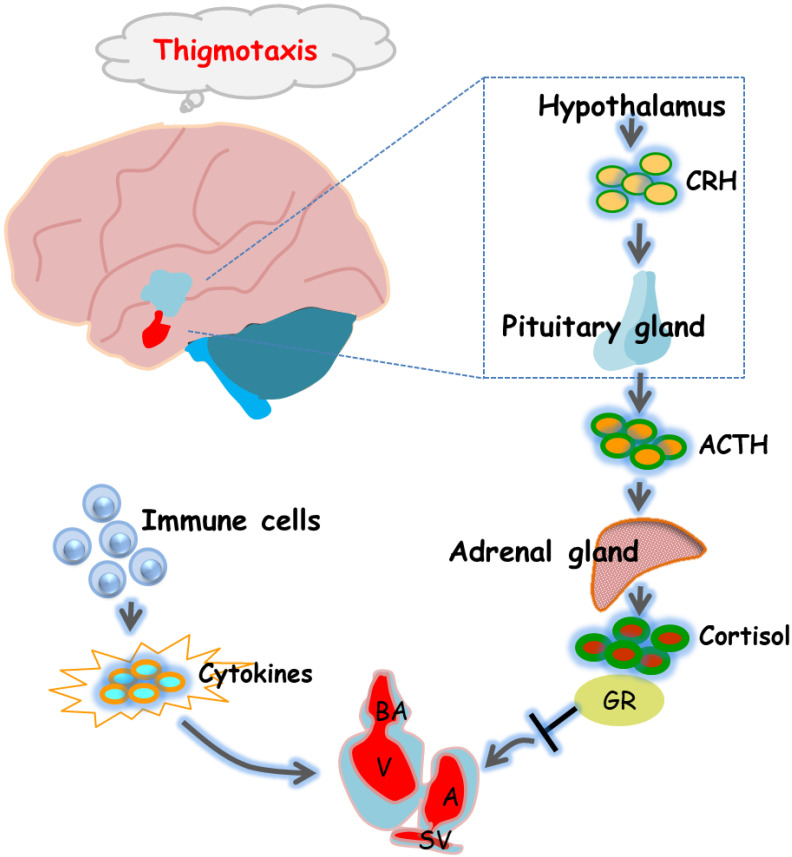
Diagram of the underlying mechanisms by which 6-BA affects zebrafish heart formation and function. 6-BA led to a defective cardiac phenotype that prevented cardiac morphogenesis, allowing abnormal cardiac cyclization and reduced cardiac contractility, thereby affecting cardiac function. In addition, mRNA levels of HPA-axis-related genes and cortisol levels differed significantly compared with controls, which was consistent with the anxious behavior observed in zebrafish at 3 dpf. Signature cytokines of the inflammatory response were also altered by 6-BA induction. In general, the study found that 6-BA induced cardiotoxicity in zebrafish, which may be related to the change of HPA axis activity and the occurrence of inflammatory response under 6-BA treatment.

The HPA axis is the main system by which animals respond to changes in their external environment and stress, and functions in a variety of physiological processes, and the HPA axis in zebrafish and humans is highly similar [33]. GR is a core molecule in the HPA axis that regulates downstream gene expression. Dysregulation of immune pathways by GR activation was found to mediate the effects of stress on morphological and functional remodeling of the developing heart, a process associated with the activation of the transcription factor Stat3 and the transcription of genes regulating cell cycle progression [8]. In addition, mouse experiments have shown that fetal heart maturation requires endogenous glucocorticoid action, and prenatal corticosteroid treatment may increase the risk of cardiovascular disease in adulthood [34]. Considering that cardiac development involves multiple complex processes, we also assessed HPA axis activity under 6-BA exposure. qPCR and hormone assay results showed that 6-BA treatment significantly increased cortisol contents and mRNA levels of HPA-axis-related genes (*crha*, *crhb*, *pomca*, *pomcb*, *nr3c1*) in zebrafish, suggesting that the HPA axis activity of larvae was altered by 6-BA induction. The HPA axis is an important aspect of the neuroendocrine system as it controls stress responses and regulates many physical activities [35]. Numerous neurotransmitters are involved in regulating hypothalamic activity, which in turn modulates activity across the HPA axis, including specific changes in mood [36]. The results obtained in behavioral experiments determined that larvae exposed to 10 and 20 mg/L 6-BA exhibited a clear thigmotaxis, indicating that 6-BA induces anxiety-like behavior. The anxiety response of zebrafish is similar to that of humans and thus can be used for the assessment of environmental toxicants, especially at small doses [37]. These behavioral changes were also associated with changes in cortisol levels, as observed in 6-BA-treated larvae: as 6-BA concentrations increased, zebrafish had higher cortisol levels and more obvious tropism. Several studies have reported correlations between systemic cortisol levels and behavioral changes in response to exposure to toxins [38,39]. Thus, the significant increase in cortisol levels in the exposed group further supports the results obtained in the thigmotaxis test that 6-BA-treated zebrafish exhibit anxiety-like behavior. Furthermore, it was found that exposure of embryos to elevated cortisol levels was involved in the repression of key genes in zebrafish heart morphogenesis, with significant adverse effects on embryonic heart morphogenesis and functional performance [40]. There is a potential link between GR-mediated cortisol signaling and cardiac morphogenesis [40]. Our observed results also further confirm that 6-BA-induced cardiotoxicity may be associated with altered HPA axis activity (Figure 6).

In conclusion, we found that acute 6-BA exposure induced cardiotoxicity in zebrafish. The experimental results showed that 6-BA caused defects in cardiac phenotype, hindered cardiac morphogenesis, and resulted in abnormal cardiac circularization and weakened cardiac function. In addition, the degree of apoptosis in zebrafish hearts was significantly increased under 6-BA treatment compared with controls, and the expression of cell-cycle-related genes was induced to decrease. At the same time, we also found that the landmark cytokines of inflammatory response were changed by 6-BA induction, and the mRNA levels of HPA-axis-related genes and cortisol content were increased under 6-BA treatment, which may contribute to 6-BA-induced cardiotoxicity. In general, the experimental results indicate that the potential toxic effects of 6-BA cannot be ignored, and the biological effects of 6-BA need to be further studied.

## 4. Materials and Methods

### 4.1. Zebrafish Rearing and Drug Treatment

Wild-type AB strains and transgenic zebrafish Tg(myl7:GFP) were purchased from the Chinese Zebrafish Resource Center and were reared in a standard recirculating aquaculture system (pH 7.0–7.2, 28.5 °C, 14 (light):10 (dark)). Zebrafish feed on freshly hatched brine shrimp twice a day. The night before the experiment, male and female zebrafish were placed in the mating box at a ratio of 1:1, separated by a transparent partition. After the partitions were removed the next morning, the males chased the females for mating. Fifteen minutes after spawning, embryos were collected in clean petri dishes, washed with fresh zebrafish water (5 mM NaCl, 0.17 mM KCl, 0.33 mM CaCl_2_, and 0.33 mM MgSO_4_, pH 7.4), and transferred to 6-well plates for culture at 28.5 °C.

6-Benzylaminopurine (purity 98%) and dimethyl sulfoxide (purity ≥ 99.8%) were purchased from Beijing Coolaber Company (Beijing, China) and Shanghai Aladdin Company (Shanghai, China), respectively. The 6-BA powder was weighed and dissolved in dimethyl sulfoxide (DMSO) solvent to make a 1 mg/mL 6-BA stock solution and stored at −20 °C protected from light. At 2 hpf, fresh system water was used to dilute the stock solutions to obtain the desired final concentrations: 10 and 20 mg/L of 6-BA solutions; system water with a DMSO ratio of 0.05% was used as the control. Subsequently, they were added to the corresponding well plates for drug exposure.

The zebrafish experimental techniques and related operations involved have been approved by the Committee for Animal Experimentation of the College of Life Science at Nankai University (no. 2008), and were carried out in strict accordance with the requirements of the NIH Guide for the Care and Use of Laboratory Animals (no. 8023, revised in 1996).

### 4.2. Morphological Observation of Early Development

According to the previous method [41,42], zebrafish developed to 48, 72, and 96 hpf were anesthetized with tricaine (0.168 g/L) and placed on the grooved slide, ensuring that the two eyes were coincident so that the zebrafish heart was clearly visible. The developmental status of zebrafish, especially the heart, was observed and photographed using a stereofluorescence microscope. The area of pericardial edema and the distance between venous sinus (sinus venosus (SV)) and arterial sinus (bulbus arteriosus (BA)) of zebrafish in different treatment groups were measured by ImageJ software, and the heart malformation rate was calculated based on the above two parameters. Each step involved was tested three times with different batches of embryos, each with 15–20 zebrafish per group.

### 4.3. Statistics of Cardiac Function Parameters

Zebrafish developed to 72 hpf were anesthetized and mounted on grooved slides to allow clear visualization of larval cardiac structures. In order to count the heart rate of zebrafish under drug treatment, a 20 s video was taken for heartbeat count and converted to beats per minute. Approximately 12–15 larvae were photographed per group. In addition, to calculate the diastolic and systolic velocities of the heart, the 10 s video recordings of the heartbeats of the larvae were taken according to the previous method [8]. The videos were analyzed frame by frame using ImageJ to calculate the end-diastolic and end-systolic areas of the heart, and the change in area over time was the diastolic or systolic velocity of the zebrafish heart.

### 4.4. Zebrafish Red Blood Cell

To assess the effect of 6-BA on cardiac hemoglobin activity, zebrafish were stained with o-dianisidine solution. Amounts 0.03 g of o-dianisidine and 0.041 g of sodium acetate were accurately weighed and placed in a 50 mL volumetric flask. After adding 1.1 mL of 35% hydrogen peroxide, the remaining volume was filled with 40% ethanol. A total of 15 zebrafish developed to 72 hpf were randomly selected from each group, stained with freshly prepared o-dianisidine solution in the dark for 15 min, and then quickly washed with DMSO solution three times. The larvae were placed side by side on a grooved glass slide, and the heart position was imaged under a stereo microscope. ImageJ was used to perform statistics on staining results for quantitative analysis. In addition, we performed video filming to analyze the blood flow rate of larvae under drug exposure, as described in the Supplementary Materials.

### 4.5. Apoptosis Assessment in Zebrafish

At 72 hpf, larvae were stained with 2.5 μg/mL of acridine orange (AO) solution for 30 min at room temperature in the dark, then washed three times with system water for 5 min each. The treated zebrafish were anesthetized with 0.168 g/L tricaine and observed under a fluorescence microscope (Olympus XZ-10, Tokyo, Japan), and whole-body and local cardiac images were collected. The cardiac fluorescence intensity of larval fish after acridine orange staining was quantitatively analyzed using Image J software (National Institute of Mental Health, Bethesda, MD, USA) to indicate the degree of cardiac apoptosis in zebrafish larvae exposed to 6-BA.

### 4.6. Determination of Cortisol Content

A total of 60 larvae that developed to 72 hpf were randomly selected from each group and placed in 1.5 mL centrifuge tubes. The weights were accurately weighed, and nine times the volume (*w*:*v*) of PBS (pH 7.4) was added for thorough homogenization. Centrifuge was at 2000–3000 rpm for about 20 min. The supernatant was carefully collected and aliquoted, one for testing and the rest frozen for later use. The experimental operation was carried out according to the instructions of the Fish Cortisol ELISA (CUSABIO, Wuhan, China). Four replicates were set up for each sample. After the measurement, the actual concentration of the sample was calculated. The experiment was repeated three times using different batches of embryos.

### 4.7. Fluorescence Quantitative PCR

Atrioventricular canal (AVC) marker genes, apoptosis-related genes, and cell cycle genes were isolated from control and different concentrations of 6-BA-exposed fish hearts in a Tg (*myl7*: EGFP) background, as previously described [43], and 60 hearts of larvae developing to 72 hpf were randomly selected from each group and placed in 1.5 mL centrifuge tubes. Total RNA was extracted using TRIzol Reagent (Leagene, Beijing, China) according to the instructions, and cDNA was synthesized by reverse transcription using a PrimeScript RT kit (Takara, Beijing, China). Real-time quantitative PCR was performed in the Multicolor Real-Time PCR Detection program using the SYBR Green labeling system. The reaction conditions were as follows: 50 °C for 2 min, 95 °C for 10 min; 95 °C for 30 s, 60 °C for 1 min, 40 cycles. Thirty larvae from each group developed to 72 hpf were extracted for total RNA and then performed real-time fluorescence quantitative PCR of other genes. The β-actin gene was used to normalize the data. The corresponding primer sequences of the target genes were listed in Table S1. Four replicates were set up for each group, and the determination of each gene was repeated three times using different batches of embryos.

### 4.8. Analysis of Thigmotaxis

The analysis of thigmotaxis behavior was performed according to previous methods [44]. A total of 30–40 larvae developed to 72 hpf were randomly selected and placed in clean 10 cm petri dishes. In a custom-made video tracking system, larvae were allowed to settle in light or dark conditions for 10 minutes to acclimate to the environment, and then the petri dish was rotated. After 30 s, the positional distribution of zebrafish in the petri dish was recorded instantly. Zebrafish that faced the dish vertically and whose head was less than 2 mm from the edge were defined as having positive thigmotaxis, and those with body contacting the wall of the dish as negative thigmotaxis larvae. Experiments were performed three times with different batches of embryos, and histogram statistics were used to analyze the effects of 6-BA exposure on early zebrafish behavior.

### 4.9. Statistical Analysis

The normal distribution of the data was assessed by the Shapiro–Wilk test. Data with a normal distribution was analyzed using one-way analysis of variance (ANOVA) and Fisher’s LSD test, and the nonparametric Kruskal–Wallis test and Dunn’s multiple comparisons test were used to analyze data that did not conform to a normal distribution. SPSS 20 (IBM Corporation, Armonk, NY, USA) and GraphPad Prism 6.0 (GraphPad Software, San Diego, CA, USA) were also used for data analysis and graphing. All data are presented as mean ± standard error (SEM). *p*-Values less than 0.05 were considered statistically significant.

## Supplementary Materials

The following supporting information can be downloaded at: https://www.mdpi.com/article/10.3390/ijms23158438/s1.
