# Supplementary material for: Cardiotoxicity of Zebrafish Induced by 6-Benzylaminopurine Exposure and Its Mechanism
Source: Int J Mol Sci. 2022 Jul 29;23(15):8438. doi: 10.3390/ijms23158438 (PMC9369308; doi:10.3390/ijms23158438)
Supplement: Supplementary file 1 [file ijms-23-08438-s001.zip › Supplementary Materials.pdf]

Supplementary materials for

Cardiotoxicity of zebrafish induced by 6-benzylaminopurine exposure and its mechanism

Authors

Mengying Yang[1], Jialu Luan[1], Yixin Xu[1], Chengtian Zhao[1], Mingzhu Sun[2,*], Xizeng Feng[1,*]

Affiliations

[1] College of Life Science, State Key Laboratory of Medicinal Chemical Biology, The Key Laboratory of Bioactive Materials, Ministry of Education, Nankai University, Tianjin 300071, China.

[2] The Institute of Robotics and Automatic Information Systems, Nankai University, Tianjin 300071, China.

*Corresponding author

E-mail: sunmz@nankai.edu.cn, xzfeng@nankai.edu.cn.

Address: Weijin Road 94, Tianjin, 300071, China. Tel: +(86) 022-23507022; Fax: +(86) 22-23507022.

1、Supplementary Method

1.1 Measurement of blood flow velocity in 72 hpf zebrafish larvae

Up to 72 hpf, the zebrafish trunk aorta blood flow video was taken under a 20-fold lens, the shooting frame rate was 100 fps, the shooting time was 1s, and about 100 images were collected for one fish. For each frame, three blood cells were tracked, and their positions in the previous frame, this frame and the next frame were recorded, including X coordinates and Y coordinates (in px), and the distance of cell flow between the two frames (in px) was calculated by the formula $\Delta s_n = \sqrt{(x_{n+1} - x_{n-1})^2 + (y_{n+1} - y_{n-1})^2}$. According to the calibration, 1px = 0.394um, the pixel value was converted into the actual flow distance (unit: um), and the actual flow velocity (unit: um/s) was calculated by the formula $\Delta d_n = \Delta s_n / 0.02$. The obtained blood flow velocity of the three cells was averaged and recorded as the blood flow velocity of the frame. In total, the blood velocity of the zebrafish was calculated for about 98 frames. The test was conducted three times independently with different batches of embryos, each time ten larvae in each group were analyzed.

2. Supplementary Figures

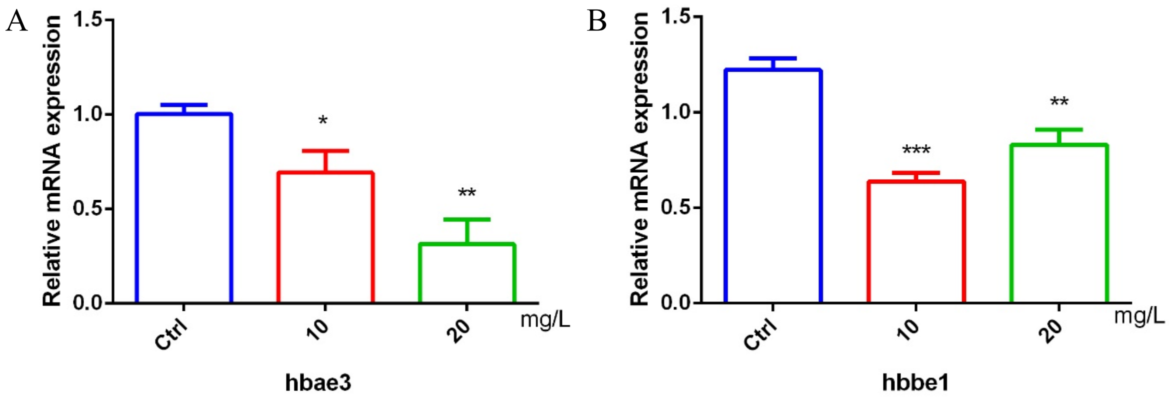

Figure S1. Abnormal embryonic hemoglobin gene expression in zebrafish exposed to 6-BA at 72 hpf. (A) Expression of the embryonic hemoglobin genes *hbae3* in zebrafish. (B) Expression of the embryonic hemoglobin genes *hbbe1* in zebrafish. Values are expressed as mean ± standard error (SEM). N=3. *p < 0.05, **p

< 0.01, ***p < 0.001, ****p < 0.0001.

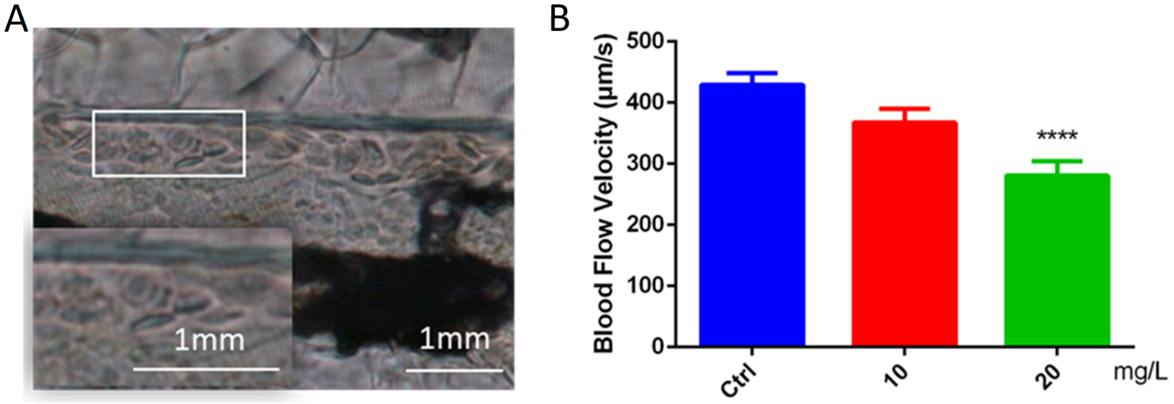

Figure S2. Effect of 6-BA on blood flow velocity of zebrafish larvae at 72 hpf. (A) Representative video image of blood flow analysis. Shown in the box were blood cells detected in the dorsal aorta. (B) The blood flow velocity of the dorsal aorta in 72 hpf larvae under different treatments. Values are expressed as mean ± standard error (SEM). N=3. *p < 0.05, **p < 0.01, ***p < 0.001, ****p < 0.0001.

3. Supplementary Table

Table S1. Sequences of primer pairs used for the real-time quantitative PCR. All sequences are shown 5'→ 3'.

Gene	Forward primer	Reverse primer
hbae3	CTCTCTCCAGGATGTTGATT	GGGACAGAATCTTGAAATTG
hbbe1	CTCTTTCCAGGACTTTGTTC	GGTTGATGATCTTGAAGTTT
myl7	GGAGAGAAGCTCAATGGCACA	GTCATTAGCAGCCTCTTGAACTCA
amhc	GCTCCTTCCTCGGTGTGAAA	TTTTCAGACTCGGCGCTCTT
vmhc	CAGTGAGGCGGTGAAAGGA A	TCTGCAGCCCTCTTGTAAGC
bmp4	CCCAGATCAAACAGGGGACC	AGGTGTTGTGCCTCACCAAA
tbx2b	AACTGGCAGAGATGCTGGTC	ACACCGGTCATTTTGGTGGT
notch1b	CGATGGTGTTGCTTAAGAATGG	ATGTGGTCTGTGATTTCCCG
bax	GTGTATGAGCGTGTTCGTC	CGGCTGAAGATTAGAGTTGT
bcl2	TGGCGTCCCAGGTAGATAAT	ACCGTACATCTCCACGAAGG
caspase3	GAGACCGCTGCCCATCACTAG	ATCCTTTCACGACCATCT
p53	CCCGGATGGAGATAACTTG	CACAGTTGTCCATTCAGCAC

ccnd1	CCAGAACCTCACCAACTTCC	TGGTCTCTGTGGAGATGTGC
ccne1	CGCAGTATGCATCAGAAAGC	TCCATAACGCGTGTATCTCG
cdk2	GATCGGAGAGGGAACATACG	GCAGAGAGATCTCACGAATGG
cdk6	GGTGCAGACTGAGGAAGAGG	TCCTGGAAACTGTGCATACG
c-myc	GGCAGCGATTCAGAAGATGAAG	CCGTCTCGTGCCTTTTCTGT
gata3	CCTGCGGACTTTACCACAAG	ACAGTTTGCGCATGAGGTC
IL-6	AGACCGCTGCCTGTCTAAAA	TTTGATGTCGTTCACCAGGA
IL1-β	TGGCGAACGTCATCCAAG	GGAGCACTGGGCGACGCATA
TNF-α	GCTTATGAGCCATGCAGTGA	TGCCCAGTCTGTCTCCTTCT
IL-10	TGG CTG AAA ATC AAG AAA GG	AGA AGA AGC GTG AGC AGA GC
IL-4	CTGTTGGTACTTACATTGGTCCCC	AGTGTCCTGTCTCATATATGTCAGGT
IL-4r	AGCAGCCAGCAGACTGAAAT	ATGGGATCGTCACAAAGTGCT
stat3	GGACTTCCCGGACAGTGAG	ATCGCTTGTGTTGCCAGAG
crha	CAGCAGACTCTCACCGACAA	CAGAGCTCCAGACGGAGAGT
crhb	CTCGCCACTTTTTGACATGA	GCTGCTCTCGATGGCTCTAC
pomca	GCTCAGTGTTGGGAAAATGC	GGTAGACGGGGGTTTCATCT
pomcb	GTGCAGATCGGACCAAGAAT	GCAAACCCAAGCTCAGACTC
nr3c1	GGCCAGTTTATGCTTTTCCA	TTGTGTGTGCCAGTCTTTCC
β-actin	AAGCAGGAGTACGATGAGT C	TGGAGTCCTCAGATGCATTG

4. Supplementary Video

Video S1. Representative videos of (A) control, (B) 10 mg/L 6-BA exposed group and (C) 20 mg/L 6-BA exposed group of 72 hpf zebrafish heart beating under fluorescence field.

S1.A: Heart fluorescence view Ctrl.mp4

S1.B: Heart fluorescence view 10.mp4

S1.C: Heart fluorescence view 20.mp4

Video S2. Representative videos of 72 hpf zebrafish cardiac systole and diastole in the original speed (A) control, (B) 10 mg/L 6-BA exposed and (C) 20 mg/L 6-BA exposed groups, as well as the slow speed (0.3×) (D) control (E) 10 mg/L 6-BA exposed and (F)

20 mg/L 6-BA exposed groups.

S2.A: Video of original velocity cardiac systole and diastole Ctrl.mp4

S2.B: Video of original velocity cardiac systole and diastole 10.mp4

S2.C: Video of original velocity cardiac systole and diastole 20.mp4

S2.D: Video of slow heart systole and diastole Ctrl.mp4

S2.E: Video of slow heart systole and diastole 10.mp4

S2.F: Video of slow heart systole and diastole 20.mp4
